# The effects of excessive and compulsive online searching of COVID-19 information (“cyberchondria”) on general and COVID-19-specific anxiety and fear in romantic couples during lockdown

**DOI:** 10.1192/j.eurpsy.2021.792

**Published:** 2021-08-13

**Authors:** S. Stewart, F. King, L. Rodriguez, S. Meier, S. Sherry, A. Abbass, H. Deacon, R. Nogueira-Arjona, A. Hagen

**Affiliations:** 1 Psychology And Neuroscience, And Psychiatry, Dalhousie University, Halifax, Canada; 2 Psychology, McGill University, Montreal, Canada; 3 Psychology, University of South Florida - St. Petersburg, St. Petersburg, United States of America

**Keywords:** cyberchondria, covid-19 fear, Anxiety, romantic couples

## Abstract

**Introduction:**

Cyberchondria involves excessive and uncontrollable online searching of information about a perceived illness. This behavior can cause or maintain distress.

**Objectives:**

Little is known about cyberchondria during the COVID-19 pandemic or how cyberchondria in one individual may cause distress in their significant other if they are self-isolating together; our study sought to fill these gaps.

**Methods:**

We conducted a Qualtrics Panel survey with 760 cohabitating Canadian couples; in June 2020, participants retrospectively reported on their cyberchondria behavior, general anxiety, and COVID-19 fears during the month of April 2020, while adhering to stay-at-home advisories. Two separate actor-partner interdependence models (APIMs) used cyberchondria excessiveness and compulsion to predict generalized anxiety and COVID-19 danger/contamination fears in the actor and partner.

**Results:**

Both cyberchondria excessiveness and compulsion were associated with higher general anxiety and higher COVID-19 danger/contamination fears in the individual (actor effects). Partner cyberchondria compulsion was associated with higher general anxiety in the individual whereas partner cyberchondria excessiveness was associated with higher COVID-19 danger/contamination fears in the individual (partner effects).

**Conclusions:**

Findings suggest that excessive and uncontrollable searching of information about COVID-19 on the internet during lockdown may contribute to distress in both the individual engaging in the cyberchondria behavior, and in their romantic partner. Moreover, different aspects of cyberchondria in the partner (compulsion vs. excessiveness) appears to contribute to general vs. COVID-19-specific anxiety/fears in the partner, respectively. Future research should examine mechanisms underlying the observed partner effects (e.g., co-rumination, social contagion) and reasons for the differential partner effects of cyberchondria components.

